# From muscle dysfunction to functionality: challenges and pathways for rehabilitation in critically ill patients

**DOI:** 10.62675/2965-2774.20260002

**Published:** 2026-09-28

**Authors:** Debora Stripari Schujmann, Raquel Annoni, Sabrina Eggmann, Saint Clair Gomes Bernardes

**Affiliations:** 1 Universidade de São Paulo Faculdade de Medicina Department of Physical Therapy São Paulo SP Brazil Department of Physical Therapy, Occupational Therapy and Speech Therapy, Faculdade de Medicina, Universidade de São Paulo - São Paulo (SP), Brazil; 2 Universidade Federal de Minas Gerais Department of Physical Therapy Belo Horizonte MG Brazil Department of Physical Therapy, Universidade Federal de Minas Gerais – Belo Horizonte (MG), Brazil; 3 University Children's Hospital Zurich Department of Intensive Care and Neonatology and Children's Research Center Zurich Switzerland Department of Intensive Care and Neonatology and Children's Research Center, University Children's Hospital Zurich, University of Zurich - Zurich, Switzerland; 4 Universidade Federal do Rio Grande do Norte Faculdade de Ciências da Saúde do Trairi Natal RN Brazil Faculdade de Ciências da Saúde do Trairi, Universidade Federal do Rio Grande do Norte - Natal (RN), Brazil

Intensive care unit-acquired weakness (ICUAW) is a debilitating and serious condition affecting approximately four out of every ten patients with a critical illness.^(1)^ It is associated with adverse events including prolonged mechanical ventilation, increased mortality, and morbidity.^(2,3)^ Survivors commonly experience physical, cognitive, mental, and social health impacts.^(2,3)^ Conversely, early mobilization has been shown to improve outcomes.^(3,4)^ The ICUAW terminology, consolidated in recent years, stems from a straightforward clinical definition: a diffuse and symmetric reduction in muscle strength. Clinicians typically diagnose it after prolonged critical illness using the Medical Research Council (MRC) sum score (< 48 points). Handgrip dynamometry serves as a diagnostic alternative, with cut-offs of < 11kg for men and < 9kg for women.^(1,2)^ As the understanding of the pathophysiology and clinical implications of ICUAW evolves, it is legitimate to question whether the term "weakness" adequately captures the complexity of this clinical picture.

Emerging evidence suggests that the answer is "no". The observed weakness may represent only the visible tip of a multifactorial iceberg of neuromuscular dysfunction, damage, and atrophy. Electrophysiological alterations, microvascular, mitochondrial, inflammatory, and metabolic dysfunctions seem to contribute to a broader clinical syndrome than the term implies^(2)^ (Figure 1). By naming the condition solely based on the symptom of weakness, there is a risk of narrowing the clinical and therapeutic focus exclusively toward strength recovery, an overly limited perspective for a syndrome with such diverse manifestations and needs.

**Figure 1:**
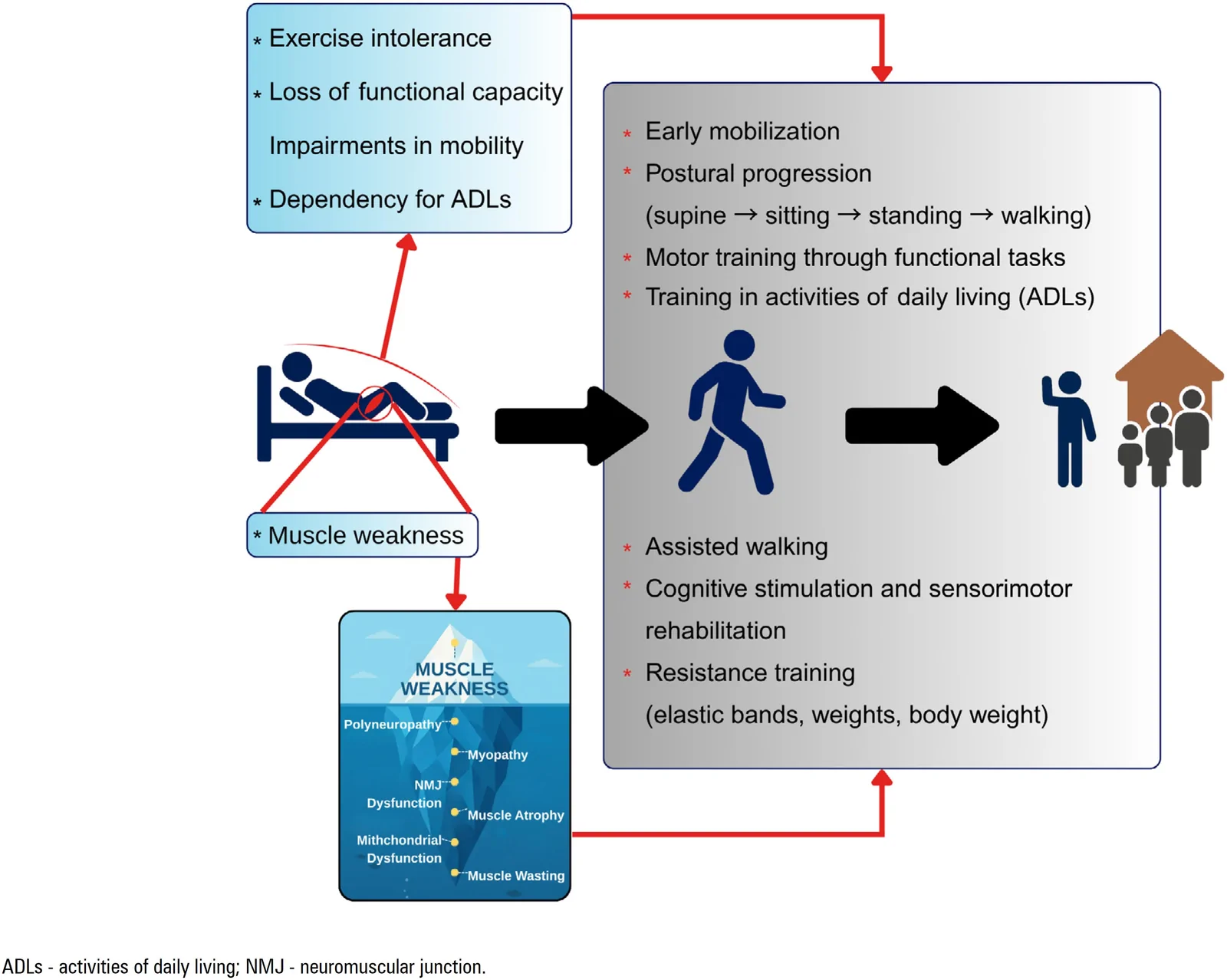
Functional pathways for early rehabilitation in critically ill patients.

Conceptual diagram illustrating how muscle weakness represents only one component of a broader functional impairment in critically ill patients. The figure highlights functional consequences such as exercises intolerance, mobility limitations, loss of functional capacity, and dependence in activities of daily living. Rehabilitation strategies extend beyond isolated strengthening and include early mobilization, progressive postural transitions (supine > sitting > standing > walking), motor training through functional tasks, assisted walking, cognitive and stimulation, and resistance training when feasible.

Figure created by the authors with the help of ChatGPT (OpenAI).

Oversimplification risks under-treatment. Muscle weakness is commonly treated with traditional resistance training, an intervention widely recommended in clinical and public health guidelines to improve muscle strength and overall health outcomes across diverse populations, such as those with coronary artery disease and chronic obstructive pulmonary disease.^(5)^ However, compared with outpatient rehabilitation settings, the implementation of such training faces substantial limitations in the intensive care unit (ICU) context. Factors such as acute inflammation, prolonged sedation, critical devices, clinical instability, inadequate nutrition, catabolism, multiple organ dysfunction, and reduced exercise tolerance pose substantial challenges to the application of traditional principles of training volume, load, and progression.

Resistance training with progressive loading is difficult to achieve in critically ill patients, and maximal loads are generally far below those of non-ICU rehabilitation programs. Patients frequently struggle to activate motor units and often fatigue before reaching effective overload.^(6)^ These limitations may be partially explained by underlying physiological alterations associated with critical illness, including intramuscular inflammation, altered metabolism, and mitochondrial dysfunction. Such disturbances compromise fatty acid oxidation, promote insulin resistance, disrupt cellular bioenergetics, and reduce adenosine triphosphate (ATP) availability.^(7)^ These bioenergetic impairments may limit the effectiveness of isolated strengthening strategies and make increases in muscle strength and mass unlikely during the early catabolic phases of critical illness.

Clinical evidence supports these physiological constraints. In a randomized controlled trial, only a minority of ICU patients could participate in progressive resistance loading.^(8)^ Furthermore, recent studies suggest that improvements in muscle structure or strength do not necessarily translate into clinically meaningful functional outcomes.^(9)^ In contrast, significant functional improvements were achieved through functional mobility interventions in the landmark trial by Schweickert et al.^(4)^ These functional gains were not accompanied by improvements in muscle strength measured by the MRC sum score or handgrip dynamometry. Together, these findings reinforce the idea that early rehabilitation in critically ill patients may benefit more from activating functional pathways and promoting motor unit recruitment than from focusing primarily on muscle mass or strength gains. Therefore, physiotherapists might need to prioritize mobility and functional tasks before attempting strengthening exercises aimed at increasing muscle mass.^(2,10)^

Perhaps the concept of ICUAW should evolve from a predominantly muscular towards a functional paradigm. Early rehabilitation should align with patients’ values, focusing on meaningful and functional activities: a core principle of person-centered care. Focusing solely on muscle strength may overlook other pathways to recovery. It is time to broaden rehabilitation interventions beyond resistance exercises and incorporate more holistic approaches.

Figure 1 illustrates a functional, pathway-oriented rehabilitation framework. Recent literature highlights the benefits of more comprehensive physical rehabilitation protocols centered on mobility and functional performance. Progressive, protocolized, and functional interventions have shown the greatest impact on clinically relevant outcomes.^(2,10-12)^ Thus, focusing on overall functional mobility has a greater impact on clinically relevant outcomes, such as time to first mobilization, time to ambulation, functional independence at hospital discharge, and functional outcomes after discharge.^(2,10-12)^ Therefore, one path is structured programs that include strategies such as in-bed activities, early mobilization, postural exercises, assisted walking, motor training through functional tasks and training of activities of daily living, cognitive stimulation, and sensorimotor rehabilitation. Notably, evidence shows that such broader interventions also contribute to reducing ICUAW at discharge.^(13,14)^ Shifting therapeutic focus beyond strength alone could therefore still positively affect this outcome. The recent TEAM trial^(15)^ suggests that more intensive early mobilization strategies are not necessarily associated with improved clinical outcomes and were associated with a higher frequency of adverse events. These findings reinforce the need for personalized exercise prescriptions that consider each patient's clinical status, cardiorespiratory and metabolic physiology, and individual characteristics. This involves shifting from "one-size-fits-all" intervention towards strategies centered on physical function and exercise capacity. A combination of multimodal components targeting diverse body structures and functions might be key to relate specific patient profiles, including post-surgical status, vasoactive drug support, and clinical severity, among others, to personalize exercise prescriptions. Ultimately, this personalization might help to balance risks and benefits according to individual physiological demands.

Rethinking the nomenclature of ICUAW goes beyond semantics and reflects how terminology shapes clinical practice. Labeling the condition as "weakness" may inadvertently limit rehabilitation approaches to strength recovery. Recognizing it as critical neuromuscular dysfunction redirects clinical reasoning toward multimodal, interdisciplinary, and functionally oriented interventions. This viewpoint advances current discussions by refining exercise prescription for ICUAW. By aligning pathophysiology, exercise principles, and clinical evidence, we move beyond generalized guidelines toward a more precise integration of patient profiles and therapeutic outcomes. This approach seeks to better balance clinical safety with individual physiological demands. Although a terminological shift requires consensus and time, we advocate that care for critically ill patients moves beyond the boundaries of muscle strength and embraces the complexity of lost functionality. Ultimately, the name of the condition matters less than how we choose to treat it.

## Data Availability

The contents underlying the research text are included in the manuscript.
